# Analysis of Free Circulating Messenger Ribonucleic Acids in Serum Samples from Late-Onset Spinal Muscular Atrophy Patients Using nCounter NanoString Technology

**DOI:** 10.3390/cells12192374

**Published:** 2023-09-28

**Authors:** Markus Leo, Linda-Isabell Schmitt, Fabian Mairinger, Andreas Roos, Christina Hansmann, Stefanie Hezel, Jelena Skuljec, Refik Pul, Ulrike Schara-Schmidt, Christoph Kleinschnitz, Tim Hagenacker

**Affiliations:** 1 Department of Neurology, Center for Translational Neuro- and Behavioral Sciences (C-TNBS), University Hospital Essen, Hufelandstr. 55, 45147 Essen, Germany; linda-isabell.schmitt@uk-essen.de (L.-I.S.); stefanie.hezel@uk-essen.de (S.H.); jelena.skuljec@uk-essen.de (J.S.); refik.pul@uk-essen.de (R.P.); christoph.kleinschnitz@uk-essen.de (C.K.); tim.hagenacker@uk-essen.de (T.H.); 2 Institute for Pathology, University Hospital Essen, Hufelandstr. 55, 45147 Essen, Germany; fabian.mairinger@uk-essen.de; 3 Department of Pediatric Neurology, Center for Neuromuscular Disorders, Center for Translational Neuro- and Behavioral Sciences (C-TNBS), University Hospital Essen, Hufelandstr. 55, 45147 Essen, Germany; andreas.roos@uk-essen.de (A.R.); ulrike.schara-schmidt@uk-essen.de (U.S.-S.)

**Keywords:** spinal muscular atrophy, survival of motor neuron, neuromuscular disorders, serum, biomarker, nanostring, targets

## Abstract

5q-related Spinal muscular atrophy (SMA) is a hereditary multi-systemic disorder leading to progressive muscle atrophy and weakness caused by the degeneration of spinal motor neurons (MNs) in the ventral horn of the spinal cord. Three SMN-enhancing drugs for SMA treatment are available. However, even if these drugs are highly effective when administrated early, several patients do not benefit sufficiently or remain non-responders, e.g., adults suffering from late-onset SMA and starting their therapy at advanced disease stages characterized by long-standing irreversible loss of MNs. Therefore, it is important to identify additional molecular targets to expand therapeutic strategies for SMA treatment and establish prognostic biomarkers related to the treatment response. Using high-throughput nCounter NanoString technology, we analyzed serum samples of late-onset SMA type 2 and type 3 patients before and six months under nusinersen treatment. Four genes (*AMIGO1*, *CA2*, *CCL5*, *TLR2*) were significantly altered in their transcript counts in the serum of patients, where differential expression patterns were dependent on SMA subtype and treatment response, assessed with outcome scales. No changes in gene expression were observed six months after nusinersen treatment, compared to healthy controls. These alterations in the transcription of four genes in SMA patients qualified those genes as potential SMN-independent therapeutic targets to complement current SMN-enhancing therapies.

## 1. Introduction

A hereditary multi-systemic disorder, 5q-related Spinal muscular atrophy (SMA) is associated with progressive muscle atrophy and weakness caused by the degeneration of motor neurons (MNs) in the ventral horn of the spinal cord [1]. SMA is classified into different phenotypes regarding its severity and onset. It is caused by homozygous deletion or compound heterozygous mutation of the *survival of motor neuron* (*SMN*) 1 gene resulting in a lack of SMN protein, which is mainly involved in the splicing process. In addition to the *SMN1*, SMN is also encoded by the *SMN2* gene, which differs from the *SMN1* in cytosine to thymine transition in exon 7, resulting in only around 10% of functional protein. In most SMN patients, the number of *SMN2* gene copies negatively correlates with the disease severity [2,3].

Recently, with nusinersen, risdiplam, and onasemnogene abeparvovec, three SMN-enhancing drugs for SMA treatment have been approved. However, even if these drugs are highly effective when administrated early, not all SMA patients benefit sufficiently. Some remain non-responders, such as adult patients suffering from late-onset SMA who have undergone a long-standing irreversible loss of MNs before starting their therapy. In these patients, a restoral that is solely SMN related has the ability to stop the progression of SMA, or motor functionality can only be slightly restored. Therefore, to improve the disease stage of patients that have not benefited from the current SMA therapies, identifying other targets to develop supporting strategies for current SMN-enhancing drugs is indispensable. Recent work suggested inflammatory pathways and cells other than neurons as potential targets, e.g., astrocytes, microglia, or muscle cells [4,5,6]. Their contribution to SMA pathogenesis is still unclear, but high-throughput screening techniques such as proteomics or transcriptomics could be a starting point for discovery studies.

Circulating nucleic acids can be detected in body fluids such as blood, cerebrospinal fluid (CSF), or urine. Therefore, they may serve as a minimally invasive tool for patient stratification regarding disease diagnosis and progression monitoring or may even contribute to identifying new therapeutic targets. For example, circulating cell-free RNAs (ccfRNAs) such as micro-RNA (miRNA) or mRNA have been identified as potential biomarkers for diagnostic and therapeutic responses in the “liquid biopsies” of cancer patients [7,8]. Moreover, recent studies have introduced an association between ccfRNAs and neurological diseases such as Amyotrophic Lateral Sclerosis (ALS) and other neurodegenerative disorders and can be potentially used as disease progression or therapy response markers [9,10,11].

To identify transcription changes in genes that could contribute to SMA pathology, serve as biomarkers, or serve as potential new therapeutic targets in circulating nucleic acid levels, we analyzed the serum samples of 30 late-onset SMA patients before and after six months of nusinersen treatment, as well as the serum samples of 10 healthy patients, using nCounter NanoString technology. Furthermore, we analyzed the gene transcription profiles of individual SMA patients in comparison to each other.

## 2. Materials and Methods

### 2.1. Serum Samples of SMA Patients and Healthy Control Individuals

Serum samples of 30 SMA patients (male and female, age 21–61 years) with 5q-SMA (type 2 or 3) and 10 age-matched healthy control individuals (male or female) were analyzed (Table 1, Supplementary Materials Table S1).

Serum was collected from SMA patients before (T0) and 6 months after the first treatment (T1) with nusinersen. SMA patients were classified by their therapeutic response as measured with the Hammersmith Functional Motor Scale Expanded (HFMSE) as (R; ≥2) or non-responder (nR).

All study participants gave written informed consent. The study was approved by the ethics committee of the University of Duisburg-Essen, Germany (approval number 18-8285-BO).

### 2.2. RNA Extraction

RNA was purified from 500 µL of patients’ serum using the Maxwell RSC miRNA Plasma or Serum kit, semi-quantitatively extracting high-quality, amplifiable total RNA, including small fragments from mammalian serum or plasma samples (Promega, Fitchburg, WI, USA). The obtained RNA was eluted in 50 µL of RNase-free water and stored at −80 °C. Before the assessment, RNA concentration was determined via Qubit Fluorometric Quantification (Thermo Fisher Science, Waltham, MA, USA), undergoing manufacturer’s instructions for the RNA broad-range assay kit. Due to the low free-RNA input generated from human serum, the maximum volume has been used as the input for the subsequent analysis.

### 2.3. nCounter CodeSet Design and Expression Analysis

The “neuropathology” nCounter panel includes a unique cell typing feature for measuring the abundance of neurons, astrocytes, microglia, oligodendrocytes, and endothelial cells. It comprises 770 genes, comprehensively assessing 23 related pathways and 30 reference genes. Hybridizations were performed using the high-sensitivity protocol on the nCounter Prep-Station. Then, post-hybridization processing was performed using the nCounter MAX/FLEX System (NanoString, Seattle, WA, USA), and the cartridge was scanned on the Digital Analyzer (NanoString, Seattle, WA, USA). Finally, the cartridge was read with maximum sensitivity (555 FOV).

### 2.4. Nanostring Data Processing

NanoString data were processed using the R statistical programming environment (v4.0.3). First, considering the counts obtained for positive control probe sets, raw NanoString counts for each gene were subjected to a technical factorial normalization. This was carried out by subtracting the mean counts plus two-times standard deviation from the CodeSet inherent negative controls. Subsequently, a biological normalization using the included RNA reference genes was performed.

Additionally, all counts with *p* > 0.05 after a one-sided *t*-test versus negative controls plus 2x standard deviations were interpreted as not expressed to overcome basal noise [12].

### 2.5. In Silico Analysis

To identify patient individual transcription differences, in silico analysis tools were used. For creating a hierarchically clustered heatmap and PCA analysis, SRplot was used. To further analyze the differentially expressed genes (DEGs) in a volcano plot, discrimination was achieved by applying a significance threshold of at least *p* < 0.05 and a minimum change of log2 fold changes > 0.5.

Gene ontology (GO) terms were identified using Cytoscape (3.9.1, open source developers) and the app BinGO (3.0.5, Ghent, Belgium). Using Cytoscape and NedREX Diamond (1.1.2, multiple developers), a network using 10 interaction partners was created. Lastly, targets for each network were identified using NedREX and the drug analysis tool.

### 2.6. Statistical Evaluation

Statistical analysis was conducted using the R i386 statistical programming environment (v4.0.2). Before exploratory data analysis, the Shapiro–Wilks test was applied to test for the normal distribution of each data set for ordinal and metric variables. Resulting dichotomous variables underwent either the Wilcoxon Mann–Whitney rank sum test (non-parametric) or two-sided student’s *t*-test (parametric).

Correlations between metrics were tested by applying Spearman’s rank correlation test and Pearson’s product–moment correlation testing for linearity.

Basic quality control of the run data was performed by mean-vs-variances plotting in order to find outliers in the target or sample levels. True differences and clusters on both target and sample levels were calculated by correlation matrix analyses.

To further specify the different candidate patterns, unsupervised and supervised clustering were performed to overcome commonalities and principal component analysis was performed to overcome differences.

## 3. Results

### 3.1. Alteration of Gene Transcription Assessed in Serum Samples of SMA Patients

**Figure 1:**
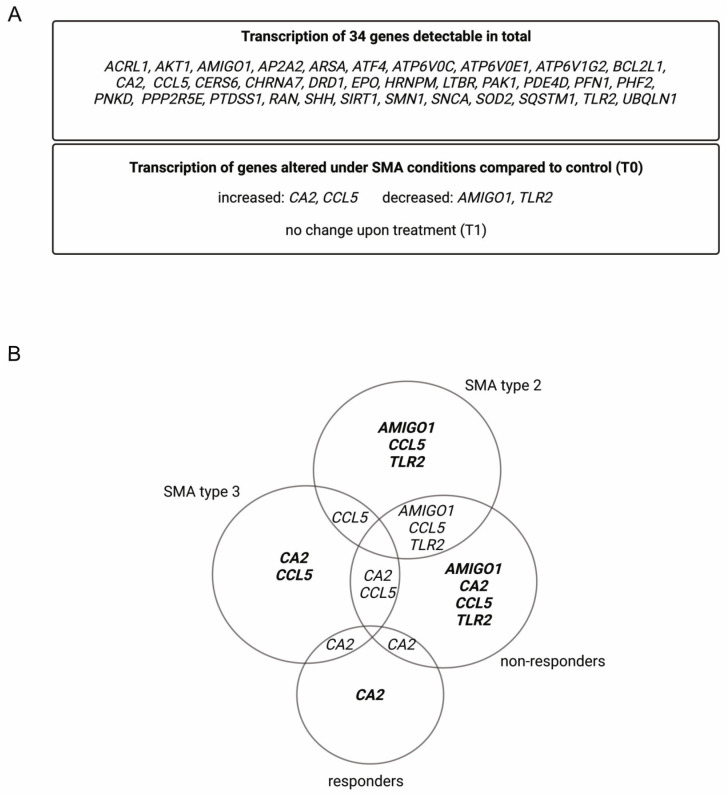
Alteration of gene transcription in serum samples of SMA patients. (**A**) Gene transcripts found in serum of SMA patients and healthy individuals. (**B**) Venn diagram of differentially transcripted genes in SMA patient subgroups.

Within the analyzed serum samples obtained from the SMA and healthy controls, transcripts of 34 genes were detectable. Four genes, Adhesion Molecule with Ig-Like Domain 1 (AMIGO1), Carbonic Anhydrase 2 (CA2), C-C Motif Chemokine Ligand 5 (CCL5), and Toll-like Receptor 2 (TLR2), showed alteration in their transcription in different SMA phenotypes at T0. CA2 and CCL5 showed enhanced transcription, while AMIGO1 and TLR2 were reduced. No change in the transcription of these genes was observed at T1 (Figure 1A).

Classifying SMA phenotypes into different subtypes revealed more specific transcription patterns. While SMA type 2 patients showed alterations in the transcription of AMIGO1, CCL5, and TLR2, in SMA type 3 patients, CA2 and CCL5 were affected. In serum from SMA R, only the transcription of CA2 was altered, whereas in SMA nR, AMIGO1, CCL5, and TLR2 were also affected (Figure 1B).

### 3.2. CA2 Transcription Is Enhanced in Serum Samples of SMA Patients

**Figure 2:**
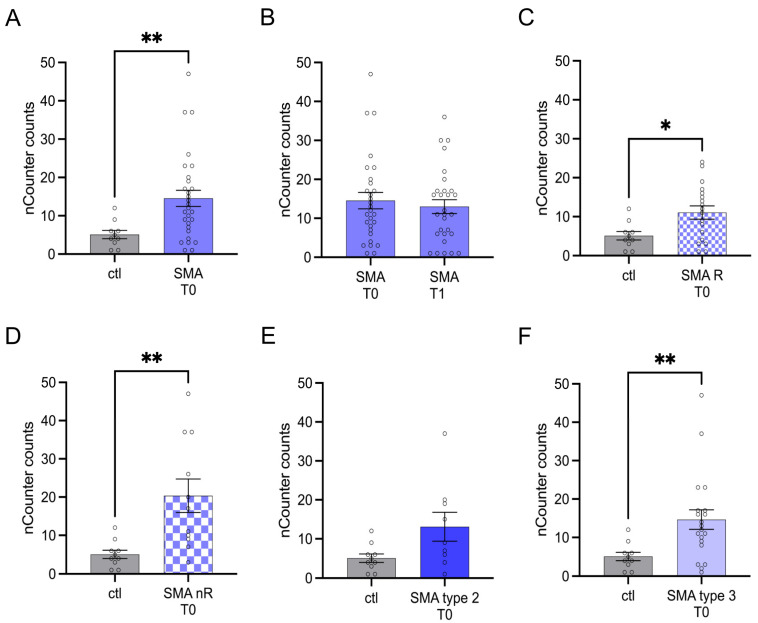
Transcription of CA2 in serum samples of SMA patients and healthy individuals. Transcription of CA2 in serum samples of SMA patients and healthy individuals. (**A**) SMA patients (total) showed enhanced CA2 transcription counts compared to healthy control individuals (*p* < 0.01). (**B**) No difference was observed when comparing the samples of SMA patients at T0 and T1 (*p* > 0.05). (**C**) CA2 transcription counts were enhanced in SMA R patients (*p* < 0.01). (**D**) Transcription counts of CA2 were enhanced in SMA nR patients (*p* < 0.05). (**E**) There was no chance of CA2 transcription in SMA type 2 patients (*p* > 0.05). (**F**) SMA type 3 patients had enhanced CA2 transcription counts (*p* < 0.01). n = 10 control individuals, 29 SMA patients (total), 18 SMA responders (R), 11 SMA non-responders (nR). Statistics: * *p* < 0.05, ** *p* < 0.01.

Serum analysis showed enhanced transcription of CA2 in SMA (total) samples compared to control at T0 (*p* < 0.01) (Figure 2A). No change was observed at T1 (*p* > 0.05) (Figure 2B).

In subtype-analysis, CA2 transcription was enhanced in both SMA R (*p* < 0.05) and SMA nR (*p* < 0.01) groups when compared to healthy controls (Figure 2C,D) at T0. CA2 was found to be enhanced in SMA type 3 patients (*p* < 0.01) at T0 (Figure 2F).

### 3.3. Transcription of CCL5 Is Enhanced in Serum Samples of SMA Type 2 and 3 Patients Who Did Not Respond to Treatment with Nusinersen

**Figure 3:**
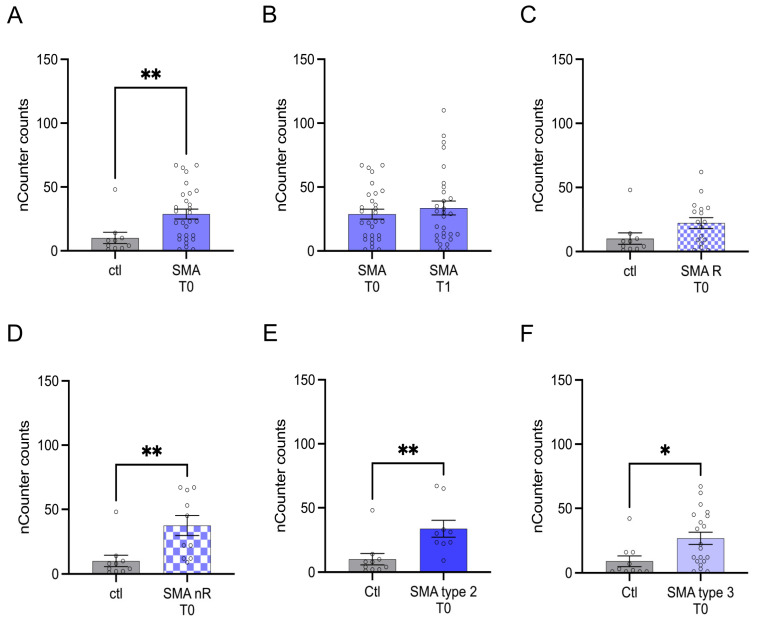
Transcription of CCL5 in serum samples of SMA patients and healthy individuals. Transcription of CCL5 in serum samples of SMA patients and healthy individuals. (**A**) SMA patients (total) showed enhanced CCL5 transcription counts compared to healthy control individuals (*p* < 0.01). (**B**) No difference was observed when comparing the samples of SMA patients at T0 and T1 (*p* > 0.05). (**C**) CCL5 transcription was unaffected in SMA R patients (*p* > 0.05). (**D**) Transcription counts of CA2 were enhanced in SMA nR patients (*p* < 0.01). (**E**) Enhanced CCL5 transcription was observed in SMA type 2 patients (*p* > 0.01). (**F**) SMA type 3 patients had enhanced CCL5 transcription counts (*p* < 0.05). n = 10 control individuals, 29 SMA patients (total), 18 SMA responders (R), 11 SMA non-responders (nR). Statistics: * *p* < 0.05, ** *p* < 0.01.

Transcription of CCL5 was enhanced in SMA (total) samples compared to control at T0 (*p* > 0.01), but no change was observed after treatment at T1 (*p* > 0.05) (Figure 3A,B).

Enhanced CCL5 transcription was detected in samples of SMA nR (*p* < 0.01) at T0 (Figure 3D). In addition, in patients suffering from SMA type 2 (*p* < 0.01) and 3 (*p* < 0.05), transcript counts of CCL5 were enhanced (Figure 3E,F).

### 3.4. AMIGO1 Transcription Is Reduced in Serum Samples of SMA nR Patients

**Figure 4:**
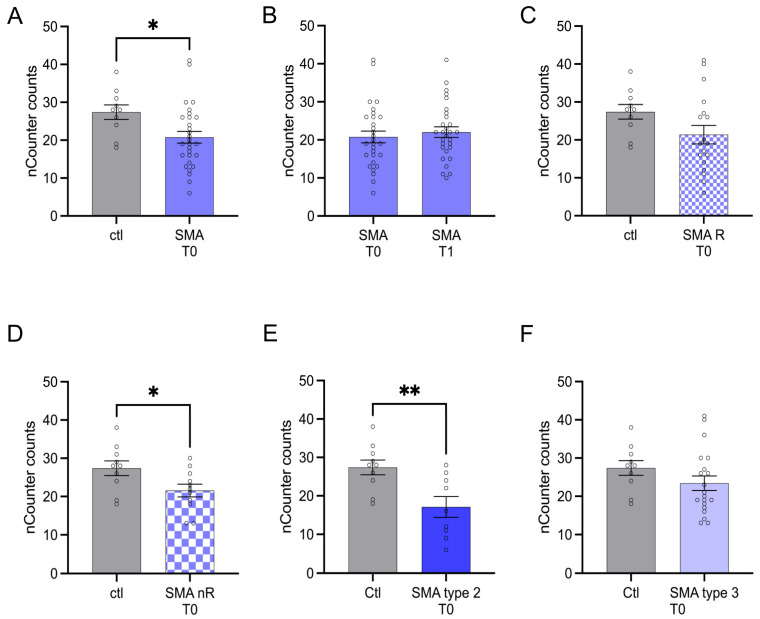
Transcription of AMIGO1 in serum samples of SMA patients and healthy individuals. Transcription of AMIGO1 in serum samples of SMA patients and healthy individuals. (**A**) SMA patients (total) showed reduced AMIGO1 transcription counts compared to healthy control individuals (*p* < 0.05). (**B**) No difference was observed when comparing the samples of SMA patients at T0 and T1 (*p* > 0.05). (**C**) AMIGO1 transcription counts were unaffected in SMA R patients (*p* > 0.05). (**D**) Transcription counts of AMIGO1 were reduced in SMA nR patients (*p* < 0.05). (**E**) Reduced AMIGO1 transcription was observed in SMA type 2 patients (*p* > 0.01). (**F**) SMA type 3 patients showed no change in AMIGO1 transcription counts (*p* > 0.05). n = 10 control individuals, 29 SMA patients (total), 18 SMA responders (R), 11 SMA non-responders (nR). Statistics: * *p* < 0.05, ** *p* < 0.01.

AMIGO1 transcription was reduced in SMA (total) patients at T0 (*p* < 0.05). No change was observed after treatment at T1 (*p* > 0.05) (Figure 4A,B).

AMIGO1 transcription was reduced in SMA nR (*p* < 0.05) at T0 (Figure 4D). In addition, a reduction of AMIGO1 transcription was detected in patients suffering from SMA type 2 (*p* < 0.01) at T0 (Figure 4E).

### 3.5. Transcription of TLR2 Was Reduced in SMA Type 2 Patients and Those Not Responding to Nusinersen Treatment

**Figure 5:**
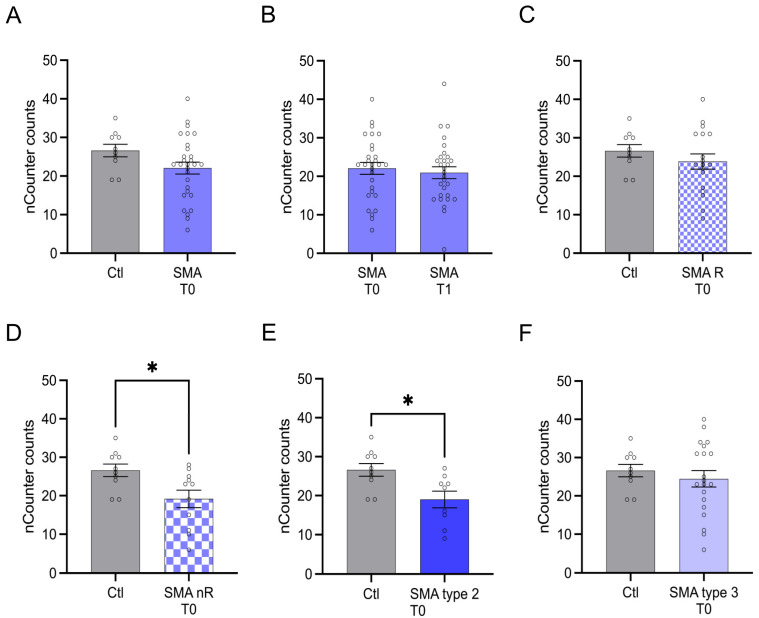
Transcription of TLR2 in serum samples of SMA patients and healthy individuals. Transcription of TLR2 in serum samples of SMA patients and healthy individuals. (**A**) SMA patients (total) showed no alteration in TLR2 transcription compared to healthy control individuals (*p* > 0.05). (**B**) No difference was observed when comparing the samples of SMA patients at T0 and T1 (*p* > 0.05). (**C**) TLR2 transcription counts were unaffected in SMA R patients (*p* > 0.05). (**D**) Transcription counts of TLR2 were reduced in SMA nR patients (*p* < 0.05). (**E**) Reduction of TLR2 transcription was observed in SMA type 2 patients (*p* < 0.05). (**F**) In SMA type 3 patients, TLR2 transcription was not affected (*p* > 0.05). n = 10 control individuals, 29 SMA patients (total), 18 SMA responders (R), 11 SMA non-responders (nR). Statistics: * *p* < 0.05.

The transcription of TLR2 did not differ between SMA (total) patients and control individuals at T0 (*p* > 0.05). Furthermore, no change in transcription was observed after treatment at T1 (*p* > 0.05) (Figure 5A,B).

In contrast, TLR2 transcription counts were reduced in SMA nR (*p* < 0.05) (Figure 5D) and SMA type 2 patients (*p* < 0.05) (Figure 5E).

### 3.6. Gene Transcript Counts Do Not Correlate with HFMSE Motor Scores

**Figure 6:**
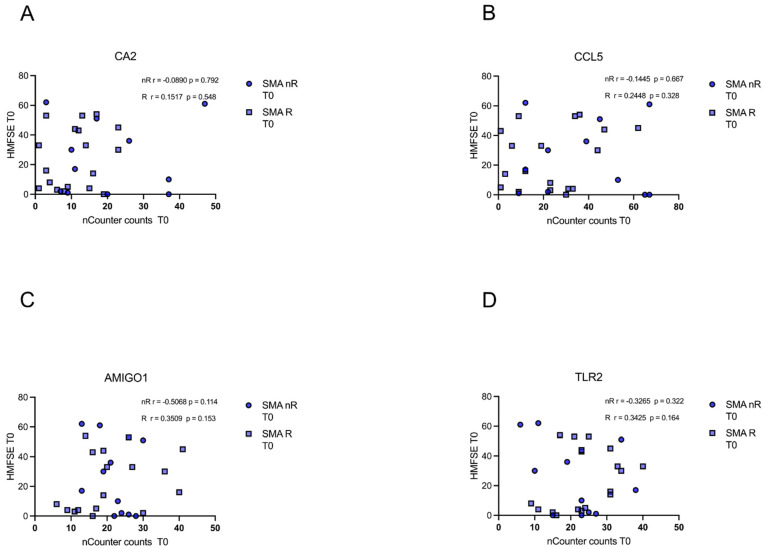
Correlation between gene transcript counts (T0) with HFMSE motor scores at T0. Correlation between gene transcript counts (T0) with HFMSE motor scores at T0. (**A**–**D**) No significant correlations were observed between gene transcript count at T0 and delta HFMSE motor scores (T0).

In either of the groups (SMA R and SMN nR), transcript counts of CA5, CCL5, AMIGO1, and TLR2 did not correlate with motor function or improvement during treatment as measured with the HFMSE (*p* > 0.05) (Figure 6A–D).

**Figure 7:**
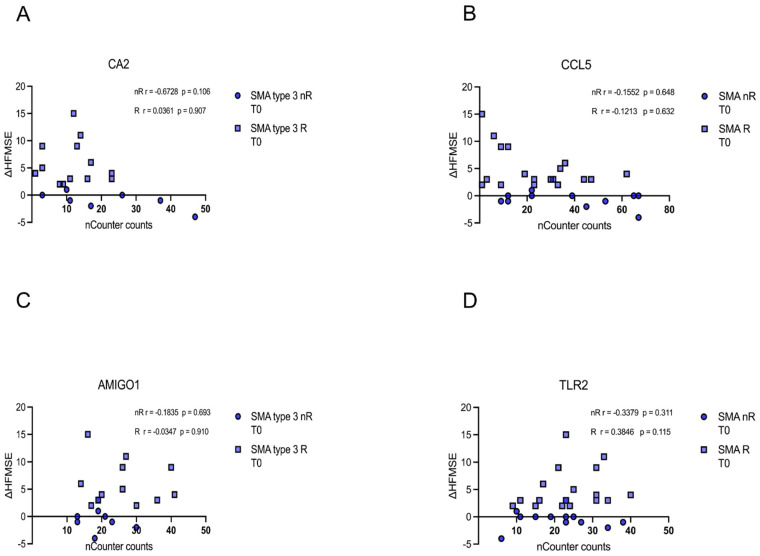
Correlation between gene transcript counts (T0) with ΔHFMSE motor scores (T0/T1). Correlation between gene transcript counts (T0) with ΔHFMSE motor scores (T0/T1). (**A**–**D**) No significant correlations were observed between gene transcript count at T0 and ΔHFMSE motor scores (T0/T1).

Similarly, no correlation of *CA5*, *CCL5*, *AMIGO1,* or *TLR5* transcript counts of each patient at T0 with the corresponding delta HFMSE motor score was found (*p* > 0.05) (Figure 7A–D).

### 3.7. Heat Map Clustering Identified Two Different Clusters within Individual Patients

**Figure 8:**
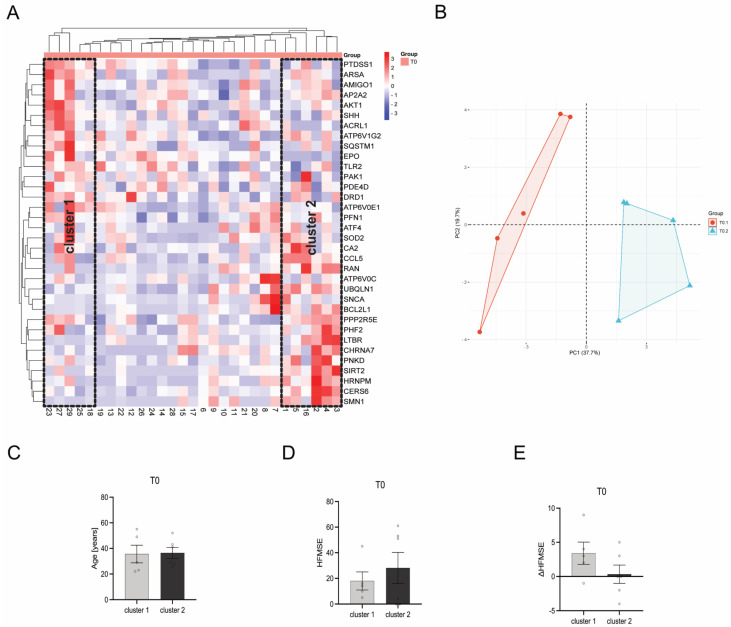
Comparison of gene transcription profiles of SMA patients at T0. (**A**) Heatmap of individual SMA patients. Two distinct clusters of SMA patients were identified after bidirectional McQuitty clustering, showing opposing gene transcription profiles. (**B**) Principal Component Analysis of SMA patients within cluster 1 and cluster 2. (**C**) Age of SMA patients in cluster 1 and cluster 2 at T0. There was no significant difference between the age of patients within the two clusters. (**D**) HFMSE motor score of SMA patients within cluster 1 and cluster 2 at T0. No statistical significance between SMA patients in cluster 1 and cluster 2 was observed, but patients within cluster 2 tended to have a higher HFMSE score. (**E**) ΔHFMSE motor score (T0 to T1) of SMA patients within cluster 1 and cluster 2. No statistical significance between the two clusters was calculated, but patients within cluster 2 tended to have a lower ΔHFMSE score.

When using a bidirectional McQuitty Clustering within the heatmap, two clusters could be identified at T0. Cluster 1 contains patients 18, 23, 25, 27, and 29, whereas cluster 2 contains patients 1, 2, 3, 4, 5, and 16 (Figure 8A,B). These clusters, upon comparative analysis of diverse patient and disease parameters such as patient age (Figure 8C), HFMSE score (Figure 8D), and ΔHFMSE score (Figure 8E), do not show any statistically significant disparities (*p* > 0.05). However, patients within cluster 2 tended to have a higher HFMSE score at T0, while ΔHFMSE scores tended to be lower than those represented by patients within cluster 1.

**Figure 9:**
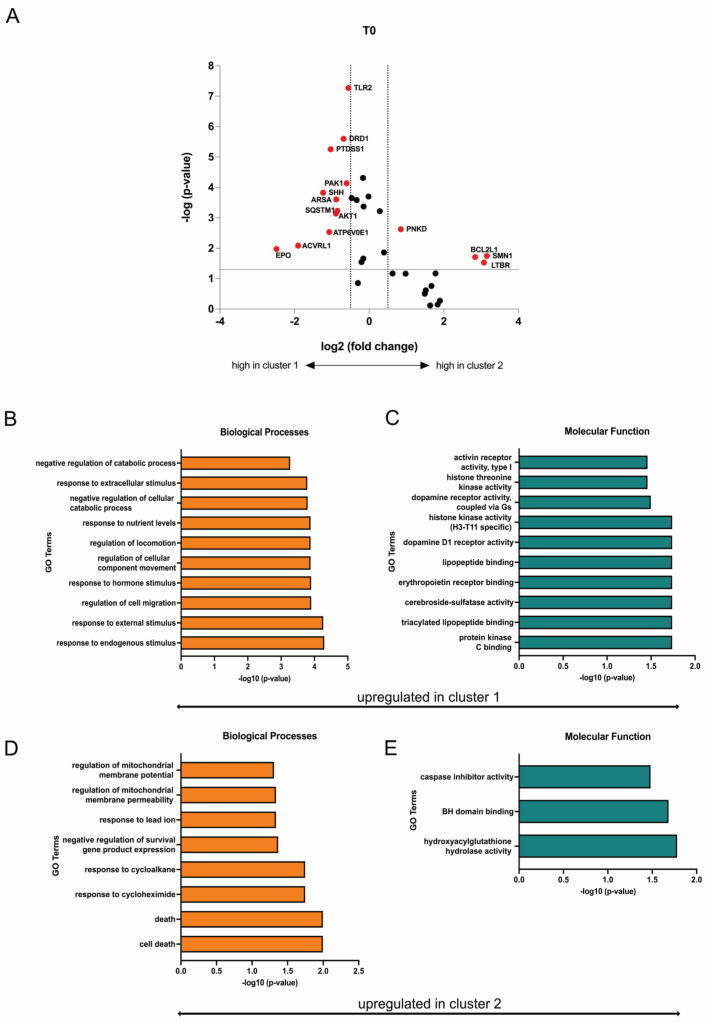
Transcriptome profiling of cluster 1 and cluster 2. (**A**) Volcano plot of DEGs (red) between cluster 1 and cluster 2. The transcription of eleven genes was enhanced in SMA patients of cluster 1, while four genes were upregulated in patients of cluster 2. Upregulated genes in each cluster were downregulated in the other one. (**B**,**C**) GO terms of upregulated genes in cluster 1 classified into biological processes and molecular function. Terms that are upregulated in cluster 1 were downregulated in Cluster 2. (**D**,**E**) GO terms of upregulated genes in cluster 2 classified into biological processes and molecular function. Terms that are upregulated in cluster 2 were downregulated in cluster 1.

Differently expressed genes (DEGs) were identified from the heatmap-based clusters through volcano plot analysis. Discrimination was achieved by applying a significance threshold of at least *p* < 0.05 and a minimum change of log2 fold changes > 0.5. Eleven genes (*EPO*, *ACVRL1*, *ATP6V0E1*, *AKT1*, *SQSTM1*, *ARSA*, *SHH*, *PAK1*, *PTDSS1*, *DRD1*, *TLR2*) were upregulated in cluster 1 and respectively downregulated in cluster 2, while four genes (*PNKD*, *BCL2L1*, *SMN1*, *LTBR*) were upregulated in cluster 2 and respectively downregulated in cluster 1 (Figure 9A). Based on the identified upregulated genes per cluster, gene ontology (GO) terms were deduced to elucidate the functions related to biological processes and molecular functions, revealing a potential increased mitochondrial function in SMA patients within cluster 2 (Figure 9B–E).

### 3.8. Network and Drug Analysis of DEGs from Cluster 1 and 2

**Figure 10:**
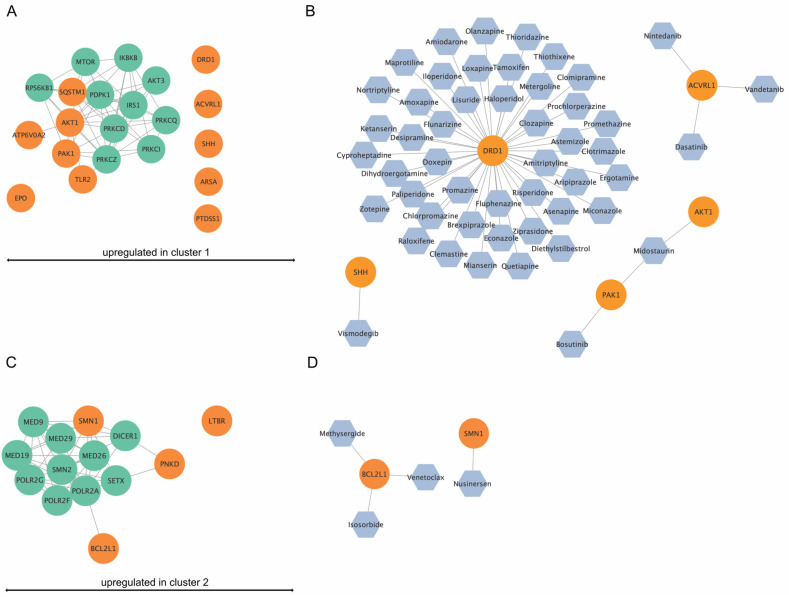
In silico network analysis and drug-finding of upregulated genes in cluster 1 and cluster 2. (**A**) Upregulated genes (orange) in cluster 1 and their top ten interaction proteins (green). *ATP6V0E1*, *PAK1*, *TLR2*, *AKT1,* and *SQSTM1* were shown to be part of an interaction network. (**B**) Drug analysis for upregulated genes in cluster 1. For *DRD1*, *SHH*, *ACVRL1*, *PAK1*, and *AKT1*, approved drugs were available. (**C**) Upregulated genes (orange) in cluster 2 and their top ten interaction proteins (green). *SMN1*, *PNKD*, and *BCL2L1* were part of an interaction network. (**D**) Drug analysis for upregulated genes in cluster 1. For *SMN1* and *BCL2L1*, approved drugs were available.

The DEGs from the various clusters are represented as networks with their top 10 interacting partners (Figure 10A,C). Furthermore, the drug analysis identified permitted drugs for upregulated genes per cluster (Figure 10B,D). For cluster 1, five of the eleven identified upregulated genes were potentially targetable by approved drugs (Figure 10B), while for cluster 2, two out of four upregulated genes were targetable by drugs (Figure 10D).

## 4. Discussion

Using a targeted approach, we analyzed free circulating mRNA transcripts in serum samples of healthy individuals or SMA patients. Those circulating mRNA transcripts are often secreted into the blood circulation by healthy and affected cells via mechanisms such as apoptosis, necrosis, and active secretion, showing disease-related variations and serving as prospective biomarkers for different clinical conditions or as potential therapeutic targets [13,14,15].

In the first step, we compared the serum mRNA transcription of clinically defined SMA subgroups (type 2, type 3, nR, R) to healthy control individuals. As a result, we found alterations in the transcription of four genes (*CA2*, *CCL5*, *AMIGO1*, *TLR2*) in SMA patients, suggesting those genes contribute to late-onset SMA pathology and could represent new potential therapeutic targets.

CA2 is one of 14 carbonic anhydrases (CA) isoforms in humans catalyzing the reversible hydration of carbon dioxide and is located in the cytoplasm [16,17,18]. CAs are involved in physiological and pathophysiological processes such as respiration, glucogenesis, or lipogenesis and are expressed among various tissues. CA2 is the most active form ubiquitously expressed in primate tissues [19]. While CA3 has been associated with neuromuscular disorders (NMD) such as Duchenne Muscular Dystrophy or Polymyositis, *CA2* has not been associated with NMD [20,21,22,23]. Here, we demonstrate the upregulation of *CA2* transcripts in the serum of SMA type 3 but not in type 2 patients, suggesting its involvement in the pathogenesis of this subgroup. At the same time, its contributing mechanism remains unclear, and further functional studies would be needed to elucidate the role of *CA2* in SMA.

CCL5 is a cytokine belonging to the C-C chemokine family. In the central nervous system (CNS), CCL5 promotes inflammation, insulin signaling, and the modulation of synaptic activity [24]. It is localized in neuroglial cells such as astrocytes, oligodendrocytes, and microglia [25,26]. Additionally, CCL5 controls the migration of blood monocytes, such as T-cells and eosinophils, from the periphery into the CNS [27]. We observed increased mRNA levels in the serum of SMA type 2 and 3 patients with a slight shift to the more severe type 2 subform.

In contrast to *CA2*, only SMA non-responders showed an increased count of *CCL5* transcripts, suggesting the involvement of inflammatory processes within this subgroup. These findings are supported by a recent study showing inflammatory signatures in the serum samples of pediatric and adult SMA patients using Bio-Plex immune assays. In addition, when patients were treated with nusinersen, not all measured cytokines were reduced to the control level (Bonanno et al., 2022).

TLR2 is a membrane receptor that recognizes pathogen-associated molecular patterns (PAMPs) derived from microorganisms [28,29]. Activation of those PAMPs results in the activation of innate immunity [29]. Here, we describe a decrease in *TLR2* transcripts in the serum of non-responding and type 2 SMA patients, while in all other subgroups, *TLR2* transcription was unaffected. Furthermore, in cultured mast cells, pro-inflammatory cytokine CCL5 was shown to reduce the expression of TLR2 [30], suggesting *TLR2* transcription downregulation in subgroups of SMA patients due to the enhanced *CCL* transcription as observed for the same patient subgroups.

In addition to *TLR2*, we detected reduced transcript counts of *AMIGO1* serum samples in the nR and type 2 patient subgroups, while in all other subgroups, *AMIGO1* was unaffected. AMIGO1 is an adhesion molecule involved in the fasciculation and myelination of developing axons [31]. In the nervous system of adults, AMIGO1 contributes to regeneration and neuronal plasticity. In addition, it is known to regulate the gating characteristics of delayed voltage-dependent potassium channels 2.1 (K_V_2.1) and 2.2 (K_V_2.2), with importance for generating action potentials and neuronal excitability [32,33,34,35]. The deletion of *AMIGO1* or the reduction of its expression leads to reduced axonal guiding and development in mice and zebrafish [36]. Such processes could contribute to the enhanced disease severity of SMA type 2 patients. Therefore, due to its role in regeneration and neuronal plasticity, the reduced transcription of *AMIGO1* in non-responding patients could prevent this group from gaining motor functionality after nusinersen treatment, assuming *AMIGO1* transcription to be crucial for therapeutic success. When comparing the findings to published gene expression data of muscle biopsies from SMA type 3 patients, none of the four presented genes were differently expressed, suggesting those secreted from the CNS or the peripheral nervous system (PNS). However, in the muscle, genes belonging to the same groups of signal transduction, transport, or cellular metabolism with similar mechanisms have been shown to be altered [37]. A micro-array study could identify regulated markers for immune response and cell cycle control in cultured muscle cells from SMA type 2 patients [38]. *CCL5*, *CA2,* and *TLR2* are also annotated with pathways and processes related to the immune system [39,40,41].

We did not find a correlation of CA2, CCL5, AMIGO1, and TLR2 transcript counts with motor function or improvement during treatment as measured with the HFMSE, suggesting these genes can not serve as predictive disease progression markers. Surprisingly, none of the genes described here were altered in their transcription exclusively in responding patients, but in non-responders as well, suggesting a distinct transcription pattern for this subgroup, defining a potential subset of prediction markers for therapeutic success. Interestingly, under SMN-enhancing treatment, none of these transcription changes were affected, suggesting those genes as potential SMN-independent targets to support SMN-enhancing therapeutic strategies. In particular, *CA2* is a likely candidate due to the availability of approved inhibitory drugs such as the anticonvulsant topiramate [42]. This needs to be addressed in further studies. However, the presented findings could contribute to a better understanding of the pathology of non-responding late-onset SMA patients. Furthermore, the data presented here also suggest that while SMA patients can be classified into different clinical phenotypes, the underlying pathomechanisms may be more individual, complicating the identification of potential biomarkers for therapeutic prediction. To overcome the current knowledge of the full complexity of SMA, studies focusing on personalized medicine are necessary.

We compared the transcription profiles of individual SMA patients under baseline conditions (T0) to identify distinct patient clusters based on genetic characteristics rather than clinically defined phenotypes. Here, we identified two clusters of SMA patients with significantly opposed gene transcription dynamics. These patients could be distinguished by their genetic profiles but did not show significant differences in age, HFMSE motor score under baseline condition, or a change in this score after nusinersen treatment. Nevertheless, patients within cluster 2 tended to have higher HFMSE scores under baseline conditions but lower ΔHFMSE scores after six months of nusinersen treatment, suggesting these patients benefit less from this therapy option than patients associated with cluster 1.

In silico network and GO term analysis showed pathways enriched in cluster 1 and cluster 2 patients, respectively. In particular, GO terms associated with mitochondria function, regulation of locomotion, cell death, or responses to endogenous, external, or extracellular stimuli are affected contrarily between patients of clusters 1 and 2. For patients within cluster 1, pathways associated with dopamine receptor signaling could be especially interesting.

In addition to network and GO term analysis, we performed in silico drug-finding, focussing on the upregulated genes in each cluster. For cluster 1, approved drugs for 5 of the 11 upregulated genes are available. Here, most drugs were found for *DRD1,* such as haloperidol, tamoxifen, or amitryptiline, suggesting *DRD1* as a potential target of interest for additional therapeutic strategies. For cluster 2, approved drugs were found for two out of four upregulated genes. Here, *BCL2L1* could be targeted by drugs such as venetoclax. 

These findings suggest an advantage of using individualized patient-based data for therapy or biomarker studies, rather than pooled SMA subtype populations. By individually monitoring each patient profile, more differences or alterations could be identified, which then leads to potentially individual-based approaches.

Our study demonstrates the use of free-circulating mRNA to pursue new avenues in SMA-patient stratification. Using in silico analysis tools, we could identify patient-individual gene transcription profiles and patient clusters based on genetics rather than clinical phenotypes. Furthermore, potential new targets and drugs were determined to complement current SMN-enhancing therapeutic strategies.

## 5. Conclusions

- There were detectable differences in gene transcript counts between the control and SMA subtype serum samples and between individual SMA patients.
- There was no alteration in gene transcript counts six months after nusinersen treatment, suggesting the SMN-independence of identified genes.
- Analysis of individual SMA patients revealed more significant insights into pathological mechanisms.
- In silico analysis can serve as a tool to identify drugs for complementing SMN-enhancing therapies.

## Figures and Tables

**Table 1:** Demographic data of the included SMA patients.

Age at Treatment, Years	Number of Patients Per Sex	SMN2 Copy Number	SMA Type	Baseline HFMSE Score	Baseline RULM Score
37 ± 13 (18–71)	female 9 (30%) male 21 (70%)	2 2 (6.6%) 3 11 (36.6%) 4 16 (53.3%) 5 1 (3.3%)	type 2 9 (30%) type 3 21 (70%)	24.46 ± 21.82	24.60 ± 13.30

HFMSE: Hammersmith Functional Motor Scale Expanded. RULM: Revised Upper Limb Module.

## Data Availability

The data supporting the study findings are available on request from the corresponding author (M.L.).

## Supplementary Materials

The following supporting information can be downloaded at: https://www.mdpi.com/article/10.3390/cells12192374/s1, Table S1: Overview of patients included in the study.
